# Unmet Family Needs in Hospice and Research Priorities: Perspectives From a National Sample of Hospice Agencies

**DOI:** 10.1093/geroni/igab046.1380

**Published:** 2021-12-17

**Authors:** Todd Becker, John Cagle

**Affiliations:** University of Maryland, Baltimore, Baltimore, Maryland, United States

## Abstract

Although hospice cares for nearly 1.5 million patients and families annually, little is known about practitioners’ opinions of current gaps in care and research. To this end, we posed two open-ended questions to hospice representatives to identify practice-relevant research priorities. Data stem from two optional questions (Q1: N = 72; Q2: N = 73) appended to Cagle et al.’s (2020) national survey of 600 randomly selected hospices, stratified by state and profit status. Most participants provided the majority of care in-home (84.7%; 79.5%) and worked at a medium-sized hospice (50.0%; 49.3%). Responses to Q1 (“What is the biggest unmet need for hospice patients and families?”) and Q2 (“In your opinion, what is the most pressing topic that hospice researchers need to study?”) were analyzed for content and then synthesized. Analyst triangulation and peer debriefing improved trustworthiness. Emerging domains included: access to hospice, hospice services and workforce issues, and education. The access to hospice domain contained a subtheme regarding the need for earlier referrals. Participants noted that short lengths of stay undermine the clinical benefits to patients and families, and that hospice enrollment criteria may contribute to inadequate lengths of stay. The hospice services and workforce issues domain largely focused on burnout prevention. Participants acknowledged that provider self-care was linked to the quality of patient care. The education domain contained subthemes related to improving physician knowledge regarding prognostication and referral, and to patients and families regarding misconceptions about hospice care. Findings highlight critical needs for future hospice research and policy change.

